# Supplementary material for: Gene-diet interactions associated with complex trait variation in an advanced intercross outbred mouse line
Source: Nat Commun. 2019 Sep 10;10:4097. doi: 10.1038/s41467-019-11952-w (PMC6736984; doi:10.1038/s41467-019-11952-w)
Supplement: Supplementary file 1 — Supplementary Information [file 41467_2019_11952_MOESM1_ESM.pdf]

Supplementary Information

for

Gene-diet interactions associated with complex trait variation in an advanced intercross outbred mouse line

Vorobyev, Gupta & Sezin et al.

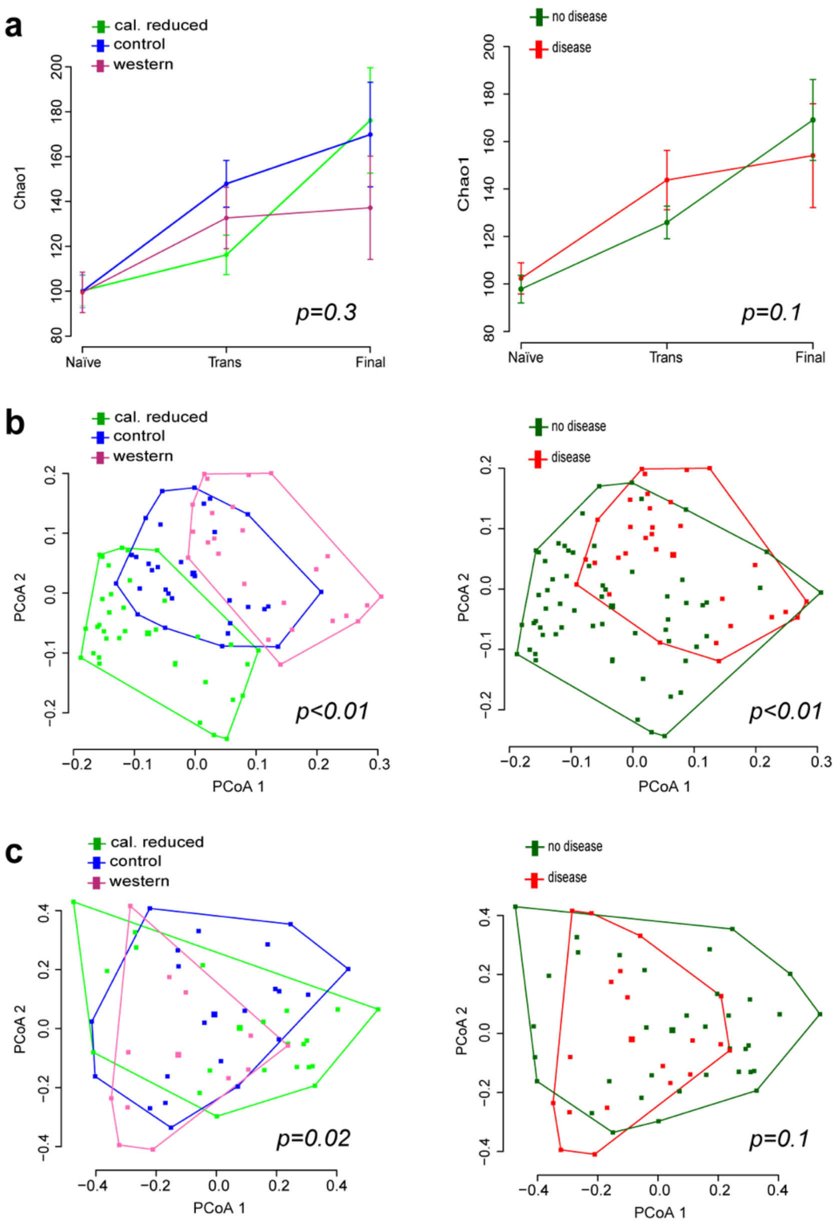

Supplementary Fig 1. Diversity of the micro- and mycobiota in NZM2410/J lupus prone mice. **a** Alpha diversity (Chao1 index) of the mycobiota between mice set on different diets across naïve, transient and final stages of disease stratified based on diet ($n_{caloric\ restriction}$=12 mice, $n_{control\ diet}$=12 mice, and $n_{western\ diet}$=10 mice, left panel) or based on the presence (n=15 mice) or absence (n=19 mice) of disease (right panel). Data is presented as mean ± SEM and statistical significance was assessed using Kruskal-Wallis test with Mann-Whitney U test as a post hoc test. **b** PCoA plots present microbial beta diversity (unweighted UniFrac distance) when the data on the microbiota are stratified based on diet ($n_{caloric\ restriction}$=18 mice, $n_{control\ diet}$=17 mice, and $n_{western\ diet}$=20 mice, left panel) or based on the presence (n=24 mice) or absence (n=31 mice) of disease (right panel) at the transient stage of disease. **c** Similarly, PCoA plots for the mycobiota (Jaccard distance) for stratification of the data based on diet ($n_{caloric\ restriction}$=11 mice, $n_{control\ diet}$=11 mice, and $n_{western\ diet}$=9 mice, left panel) or disease presence (n=13 mice) and absence (n=18 mice) (right panel) in the transient stage are shown. Data were assessed using the adonis function in R (permutations=999) by comparing centroids. Source data for (a-c) are provided as a Source Data file.

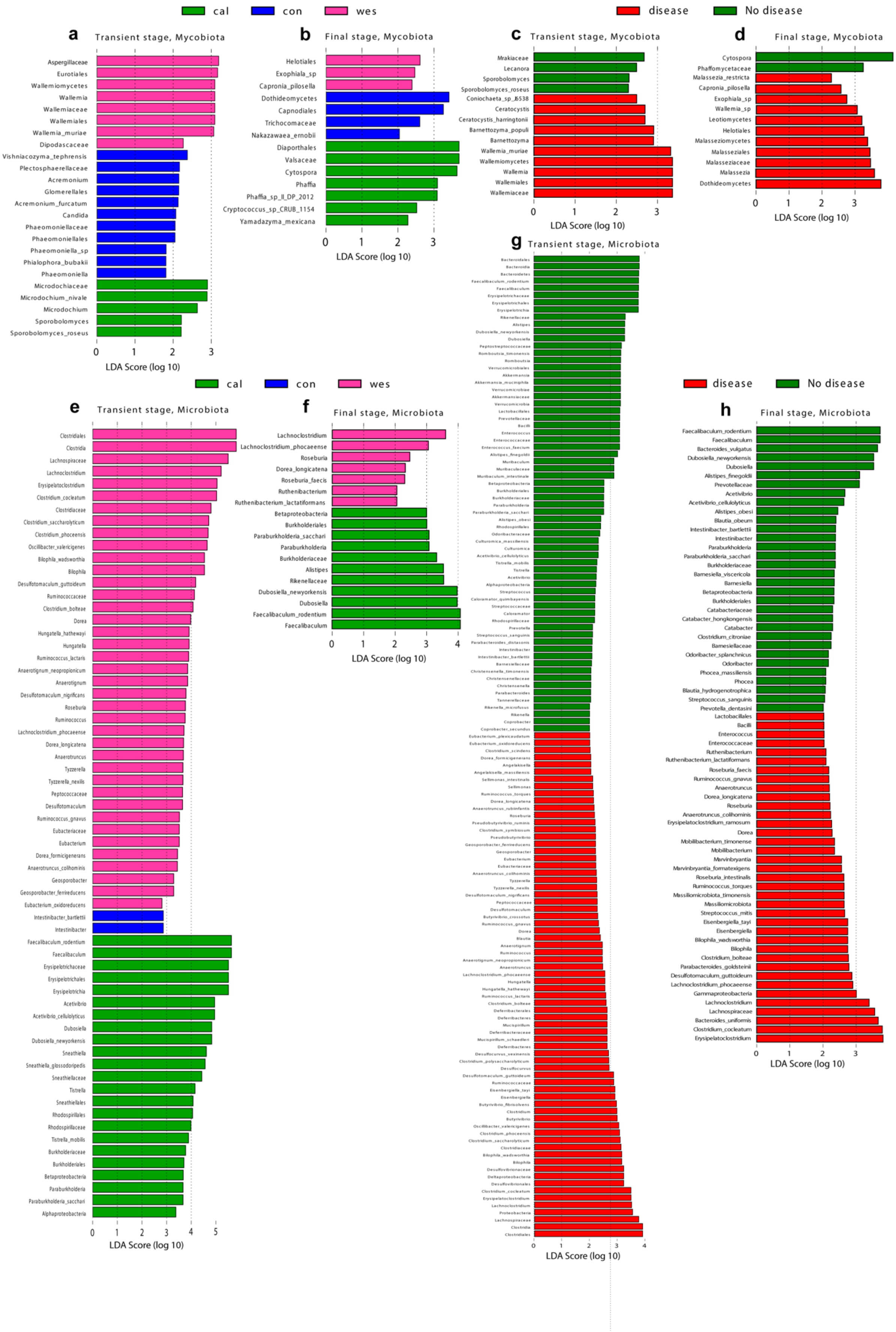
cal
con
wes
disease
No disease
a
Transient stage, Mycobiota
Aspergillaceae
Eurotiales
Wallemiomycetes
Wallemia
Wallemiaceae
Wallemiales
Wallemia_muriae
Dipodascaceae
Vishniacozyma_tephrensis
Plectosphaerellaceae
Acremonium
Glomerellales
Acremonium_furcatum
Candida
Phaeomoniellaceae
Phaeomoniellales
Phaeomoniella_sp
Phialophora_bubakii
Phaeomoniella
Microdochiaceae
Microdochium_nivale
Microdochium
Sporobolomyces
Sporobolomyces_roseus
LDA Score (log 10)
b
Final stage, Mycobiota
Helotiales
Exophiala_sp
Capronia_pilosella
Dothideomycetes
Capnodiales
Trichocomaceae
Nakazawaea_ernobii
Diaporthales
Valsaceae
Cytospora
Phaffia
Phaffia_sp_II_DP_2012
Cryptococcus_sp_CRUB_1154
Yamadazyma_mexicana
LDA Score (log 10)
c
Transient stage, Mycobiota
Mrakiaceae
Lecanora
Sporobolomyces
Sporobolomyces_roseus
Coniochaeta_sp_J6538
Ceratocystis
Ceratocystis_harringtonii
Barnettozyma_populi
Barnettozyma
Wallemia_muriae
Wallemiomycetes
Wallemia
Wallemiales
Wallemiaceae
LDA Score (log 10)
d
Final stage, Mycobiota
Cytospora
Phaffomycetaceae
Malassezia_restricta
Capronia_pilosella
Exophiala_sp
Wallemia_sp
Leotiomycetes
Helotiales
Malasseziomycetes
Malasseziales
Malasseziaceae
Malassezia
Dothideomycetes
LDA Score (log 10)
g
Transient stage, Microbiota
LDA Score (log 10)
e
Transient stage, Microbiota
Clostridiales
Clostridia
Lachnospiraceae
Lachnoclostridium
Erysipelatoclostridium
Clostridium_cocleatum
Clostridiaceae
Clostridium_saccharolyticum
Clostridium_phocaeensis
Oscillibacter_valericigenes
Bilophila_wadsworthia
Bilophila
Desulfotomaculum_guttoideum
Ruminococcaceae
Clostridium_bolteae
Dorea
Hungatella_hathewayi
Hungatella
Ruminococcus_lactaris
Anaerotignum_neopropionicum
Anaerotignum
Desulfotomaculum_nigrificans
Roseburia
Ruminococcus
Lachnoclostridium_phocaeense
Dorea_longicatena
Anaerotruncus
Tyzzerella
Tyzzerella_nexilis
Peptococcaceae
Desulfotomaculum
Ruminococcus_gnavus
Eubacteriaceae
Eubacterium
Dorea_formicigenerans
Anaerotruncus_colihominis
Geosporobacter
Geosporobacter_ferrireducens
Eubacterium_oxidoreducens
Intestinibacter_bartlettii
Intestinibacter
Faecalibaculum_rodentium
Faecalibaculum
Erysipelotrichaceae
Erysipelotrichales
Erysipelotrichia
Acetivibrio
Acetivibrio_cellulolyticus
Dubosiella
Dubosiella_newyorkensis
Sneathiella
Sneathiella_glossodoripedis
Sneathiellaceae
Tistrella
Sneathiellales
Rhodospirillales
Rhodospirillaceae
Tistrella_mobilis
Burkholderiaceae
Burkholderiales
Betaproteobacteria
Paraburkholderia
Paraburkholderia_sacchari
Alphaproteobacteria
LDA Score (log 10)
f
Final stage, Microbiota
Lachnoclostridium
Lachnoclostridium_phocaeense
Roseburia
Dorea_longicatena
Roseburia_faecis
Ruthenibacterium
Ruthenibacterium_lactatiformans
Betaproteobacteria
Burkholderiales
Paraburkholderia_sacchari
Paraburkholderia
Burkholderiaceae
Alistipes
Rikenellaceae
Dubosiella_newyorkensis
Dubosiella
Faecalibaculum_rodentium
Faecalibaculum
LDA Score (log 10)
h
Final stage, Microbiota
Faecalibaculum_rodentium
Faecalibaculum
Bacteroides_vulgatus
Dubosiella_newyorkensis
Dubosiella
Alistipes_finegoldii
Prevotellaceae
Acetivibrio
Acetivibrio_cellulolyticus
Alistipes_obesi
Blautia_obeum
Intestinibacter_bartlettii
Intestinibacter
Paraburkholderia
Paraburkholderia_sacchari
Burkholderiaceae
Barnesiella_viscericola
Barnesiella
Betaproteobacteria
Burkholderiales
Catabacteriaceae
Catabacter_hongkongensis
Catabacter
Clostridium_citroniae
Barnesiellaceae
Odoribacter_splanchnicus
Odoribacter
Phocea_massiliensis
Phocea
Blautia_hydrogenotrophica
Streptococcus_sanguinis
Prevotella_dentasini
Lactobacillales
Bacilli
Enterococcus
Enterococcaceae
Ruthenibacterium
Ruthenibacterium_lactatiformans
Roseburia_faecis
Ruminococcus_gnavus
Anaerotruncus
Dorea_longicatena
Roseburia
Anaerotruncus_colihominis
Erysipelatoclostridium_ramosum
Dorea
Mobilibacterium_timonense
Mobilibacterium
Marvinbryantia
Marvinbryantia_formatexigens
Roseburia_intestinalis
Ruminococcus_torques
Massiliomicrobiota_timonensis
Massiliomicrobiota
Streptococcus_mitis
Eisenbergiella_tayi
Eisenbergiella
Bilophila_wadsworthia
Bilophila
Clostridium_bolteae
Parabacteroides_goldsteinii
Desulfotomaculum_guttoideum
Lachnoclostridium_phocaeense
Gammaproteobacteria
Lachnoclostridium
Lachnospiraceae
Bacteroides_uniformis
Clostridium_cocleatum
Erysipelatoclostridium
LDA Score (log 10)

Supplementary Fig 2. LEfSe bar plots of micro- and mycobiotic taxonomic biomarkers for different diets and based on presence/absence of disease. The features are plotted as color-coded bars on a logarithmic scale of linear discriminant analysis (LDA) scores. The features were considered statistically significant if $\log_{10}$ of the LDA score > 1.5 and if the Kruskal-Wallis test (for diet) or Mann-Whitney U test (for disease) yields a P-value < 0.05. Bar plots in (**a**) and (**b**) show LDA scores for three diets for the mycobiome at the transient and final stages of disease, respectively. Bar plots in (**c**) and (**d**) depict LDA scores for diseased versus non-diseased mice for the mycobiome at the transient and final stages of disease, respectively. Similarly, LDA scores in bar plots (**e**) and (**f**) are shown for stratification of the microbiota data based on diets in the transient and final stages, respectively. LDA scores for microbiota data in diseased versus non-diseased mice in the transient (**g**) and final stages (**h**) of disease are shown. Source data for (a-h) are provided as a Source Data file.

a

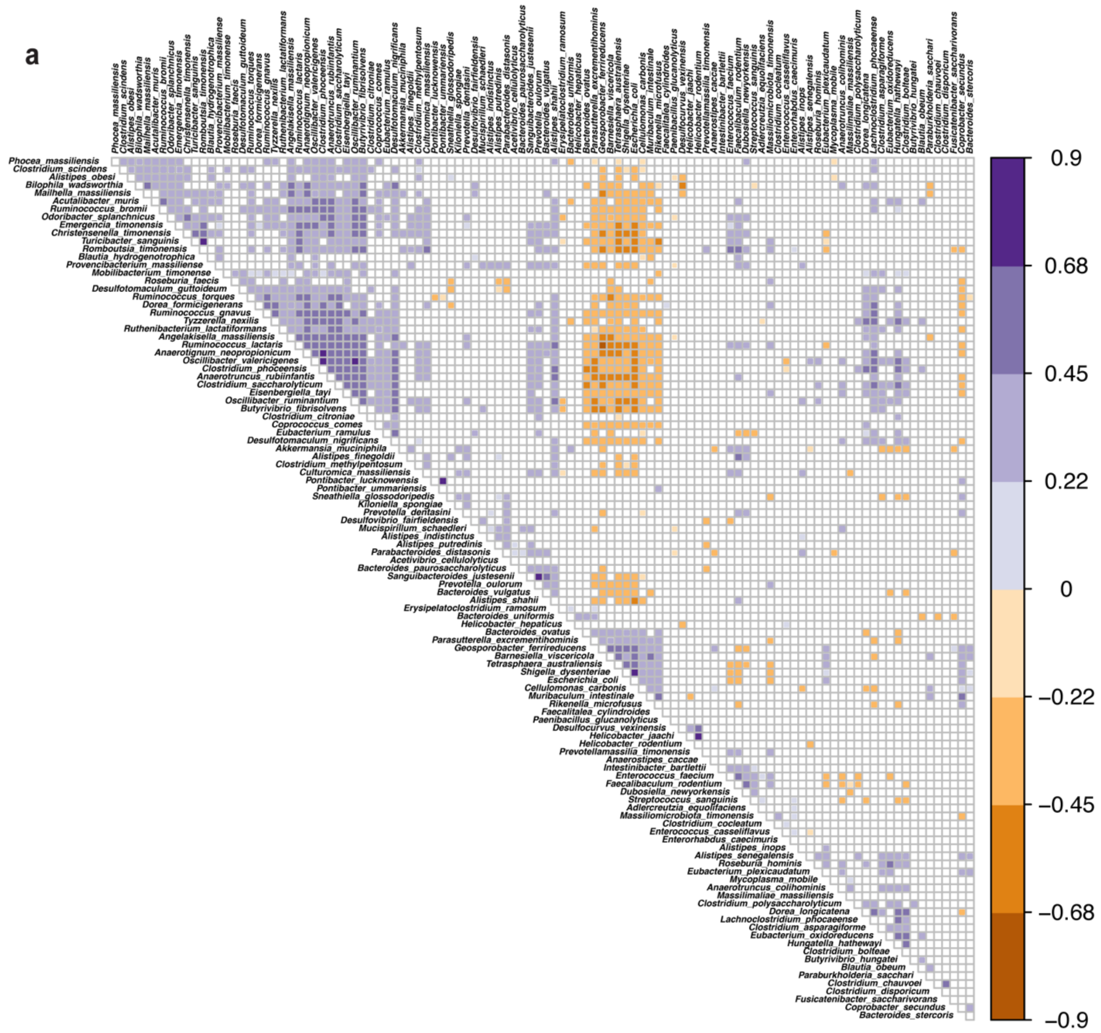

b

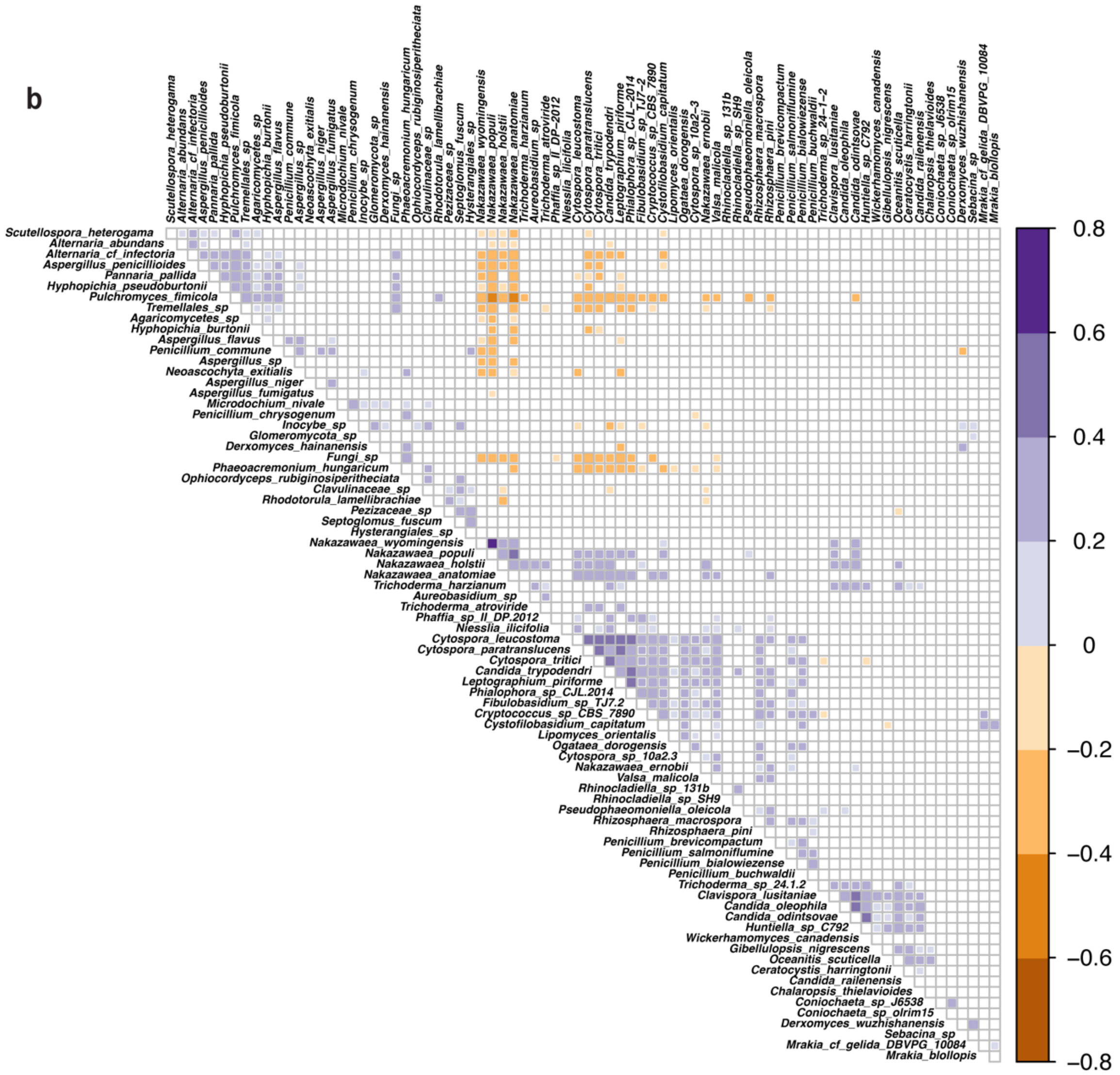

Scutellospora_heterogama
Alternaria_abundans
Alternaria_cf_infectoria
Aspergillus_penicillioides
Pannaria_pallida
Hyphopichia_pseudoburtonii
Pulchromyces_fimicola
Tremellales_sp
Agaricomycetes_sp
Hyphopichia_burtonii
Aspergillus_flavus
Penicillium_commune
Aspergillus_sp
Neoascochyta_exitialis
Aspergillus_niger
Aspergillus_fumigatus
Microdochium_nivale
Penicillium_chrysogenum
Inocybe_sp
Glomeromycota_sp
Derxomyces_hainanensis
Fungi_sp
Phaeoacremonium_hungaricum
Ophiocordyceps_rubiginosiperitheciata
Clavulinaceae_sp
Rhodotorula_lamellibrachiae
Pezizaceae_sp
Septoglomus_fuscum
Hysterangiales_sp
Nakazawaea_wyomingensis
Nakazawaea_populi
Nakazawaea_holstii
Nakazawaea_anatomiae
Trichoderma_harzianum
Aureobasidium_sp
Trichoderma_atroviride
Phaffia_sp_II_DP.2012
Niesslia_ilicifolia
Cytospora_leucostoma
Cytospora_paratranslucens
Cytospora_tritici
Candida_trypodendri
Leptographium_piriforme
Phialophora_sp_CJL.2014
Fibulobasidium_sp_TJ7.2
Cryptococcus_sp_CBS_7890
Cystofilobasidium_capitatum
Lipomyces_orientalis
Ogataea_dorogensis
Cytospora_sp_10a2.3
Nakazawaea_ernobii
Valsa_malicola
Rhinocladiella_sp_131b
Rhinocladiella_sp_SH9
Pseudophaeomoniella_oleicola
Rhizosphaera_macrospora
Rhizosphaera_pini
Penicillium_brevicompactum
Penicillium_salmoniflumine
Penicillium_bialowiezense
Penicillium_buchwaldii
Trichoderma_sp_24.1.2
Clavispora_lusitaniae
Candida_oleophila
Candida_odintsovae
Huntiella_sp_C792
Wickerhamomyces_canadensis
Gibellulopsis_nigrescens
Oceanitis_scuticella
Ceratocystis_harringtonii
Candida_railenensis
Chalaropsis_thielavioides
Coniochaeta_sp_J6538
Coniochaeta_sp_olrim15
Derxomyces_wuzhishanensis
Sebacina_sp
Mrakia_cf_gelida_DBVPG_10084
Mrakia_blollopis
0.8
0.6
0.4
0.2
0
−0.2
−0.4
−0.6
−0.8

c

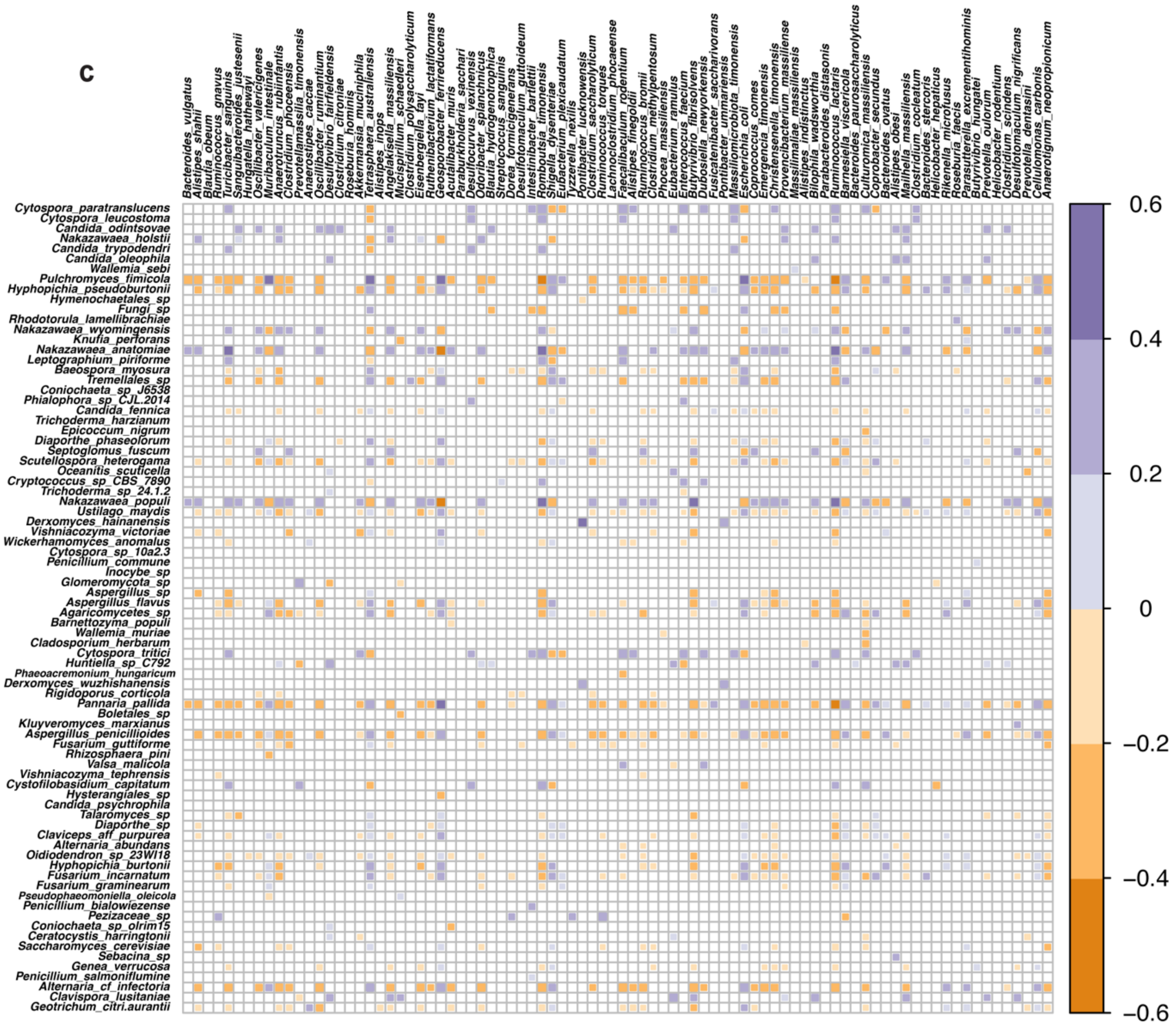

Supplementary Fig 3. **a** Correlation triangular plot showing a correlation between the microbial OTUs (species identified by both RDP and NCBI BLAST) calculated by the SparCC algorithm (FastSpar R package). The color code and its depth inside the cells show positive (purple) and negative (orange) correlation among the species (Padj < 0.05, Benjamini-Hochberg correction). **b** Correlation triangular plot showing a correlation between the fungal OTUs (species identified by both RDP and NCBI BLAST) calculated by the SparCC algorithm (FastSpar R package). The color code and its depth inside the cells show positive (purple) and negative (orange) correlation among the species (Padj < 0.05, Benjamini-Hochberg correction). **c** Heatmap showing correlation between mycobiotic and microbial OTUs (species identified by both RDP and NCBI BLAST) calculated by the SparCC algorithm (FastSpar R package). The color code and its depth inside the cells show positive (purple) and negative (orange) correlation among the species (Padj < 0.05, Benjamini-Hochberg correction). Source data for (a-c) are provided as a Source Data file.

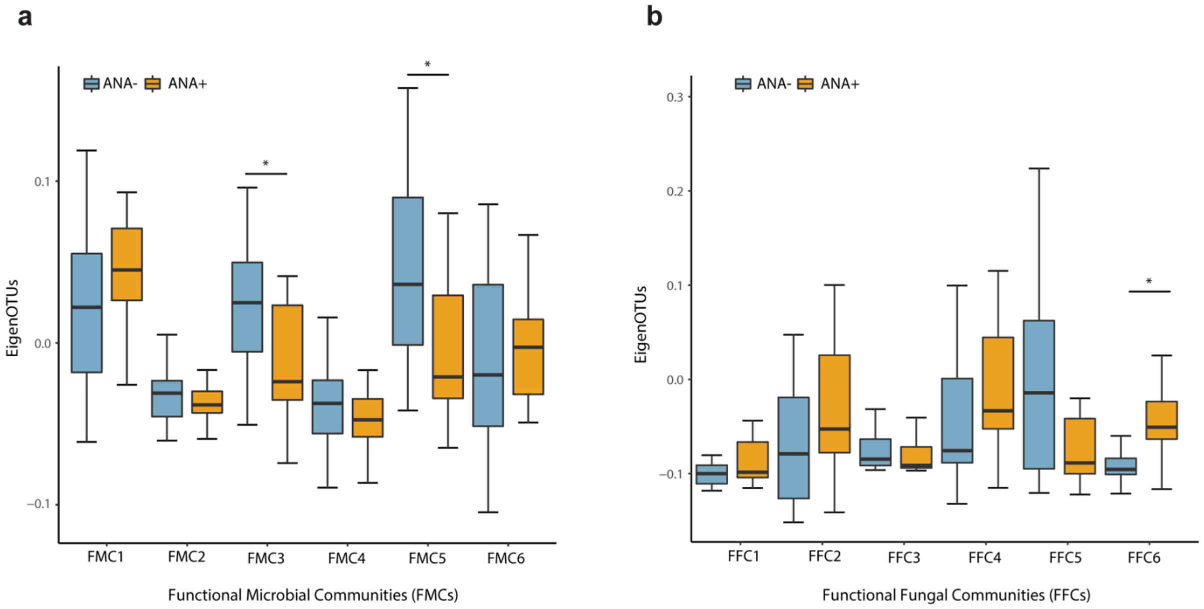

Supplementary Fig 4. Boxplot (the band indicates the median, the box indicates the first and third quartiles and whiskers indicate 1.5*interquartile range) showing the eigengene values on the y-axis and functional communities of bacteria (**a**) and fungi (**b**) on the x-axis, stratified based on the absence (n_{FMC} = 37 mice, n_{FFC} = 18 mice) or presence (n_{FMC} = 16 mice, n_{FFC} = 16 mice) of antinuclear antibody in NZM2410/J mice. Statistical significance was assessed using the Mann-Whitney U test and adjusted for multiple testing by the Benjamini-Hochberg correction. *Padj < 0.05. Source data for (a,b) are provided as a Source Data file.

Phenotypes	Unit	Assessment method	N	σ sex (%)	σ diet (%)
Physiological phenotypes					
Metabolic phenotypes					
Body weight (final)	g	Weighting at 6 months	1,064	5.52	47.94
- body weight (month 2)	*g*	Weighting at 2 months	*777*	8.07	33.67
- body weight (month 4)	*g*	Weighting at 4 months	*779*	7.25	45.5
Spleen weight	g	Weighting at 6 months	1,065	0.81	14.21
Total cholesterol	mg/dl	ELISA	853	0.61	14.75
HDL[1] cholesterol	mg/dl	ELISA	835	1.06	13.14
LDL cholesterol	mg/dl	ELISA	853	0.1	8.75
Hematological phenotypes					
WBC	K/µl	HemaVet	467	1.42	1.13
Neutrophils	K/µl	HemaVet	467	0.12	0.21
Lymphocytes	K/µl	HemaVet	467	1.66	2.68
Monocytes	K/µl	HemaVet	467	2.45	0.34
Eosinophils	K/µl	HemaVet	467	2.18	4.22
Basophils	K/µl	HemaVet	467	1.2	1.69
Neutrophils / WBC	%	HemaVet	467	0.48	3.4
Lymphocytes / WBC	%	HemaVet	467	0.05	4.55
Monocytes / WBC	%	HemaVet	467	0.97	0.09
Eosinophils / WBC	%	HemaVet	467	1.44	5.73
Basophils / WBC	%	HemaVet	467	2.47	3.4
RBC	M/µl	HemaVet	467	0.99	0.08
Hb	g/dL	HemaVet	467	0.48	0.07
HCT	%	HemaVet	467	0.2	0.64
MCV	fL	HemaVet	467	0.07	1.93
MCH	pg	HemaVet	467	0.17	0.03
MCHC	g/dL	HemaVet	467	0	2.43
RDW	%	HemaVet	467	3.19	3.42
Platelets	K/µl	HemaVet	467	1.72	1.97
MPV	fL	HemaVet	467	0.38	1.44
Immunoglobulins					
IgA serum concentration	mg/ml	ELISA	534	0.55	19.14
IgG serum concentration	mg/ml	ELISA	534	4.1	2.11
IgM serum concentration	mg/ml	ELISA	534	0.69	26.19
IgA x G x M serum concentration	mg/ml	ELISA	534	0.46	0.19
(IgA x IgG) / IgM	-	ELISA	534	0.07	28.63
IgA x IgG	-	ELISA	534	0.06	9.08
IgA / IgG	-	ELISA	534	2.96	24.58
IgA / IgM	-	ELISA	534	1.02	37.02
IgG / IgM	-	ELISA	534	0.17	18.74
Glycosylation patterns					
IgG – G0	%	HILIC-HPLC	699	0.46	6.72
IgG – G1	%	HILIC-HPLC	699	0.65	12.53
IgG – G2	%	HILIC-HPLC	699	0.27	4.22

IgG - G1S1	%	HILIC-HPLC	699	0.59	5.24
IgG – G2S1	%	HILIC-HPLC	699	0.13	0.1
IgG – G2S2	%	HILIC-HPLC	699	0.56	0.77
IgG – presence of terminal galactose	%	HILIC-HPLC	699	0.67	12.45
IgG – total galactosylated	%	HILIC-HPLC	699	0.46	6.72
IgG – monogalactosylated	%	HILIC-HPLC	699	0.42	9.84
IgG – bigalactosylated	%	HILIC-HPLC	699	0.22	1.17
IgG – total sialylated	%	HILIC-HPLC	699	0	0.38
IgG sialylated/galactosylated	-	HILIC-HPLC	699	0.08	4.96
Others					
Coat colour	Binary	Visual	1,154	0.06	0
Pathophysiological phenotypes					
CRP	pg/ml	ELISA	435	1.64	14.56
ANA	Binary	IF on Hep-20-10 cells	1,113	0.57	4.28
NAFLD	Score	assessment of H&E stained sections	653	1.63	41.41
Balloning	*As above*	assessment of H&E stained sections	653	3.43	46.43
Steatosis	*As above*	assessment of H&E stained sections	653	0.4	28.28
NASH	*As above*	assessment of H&E stained sections	653	0.09	11.63

- "N" indicates number of mice

- Abbreviations: HDL: high-density lipoproteins; LDL: low-density lipoprotein; CRP: C-reactive protein; WBC: white blood cells; RBC: red blood cells; Hb: haemoglobin; MCV: mean corpuscular volume; MCH: mean corpuscular haemoglobin; MCHC: mean corpuscular haemoglobin concentration; RDW: red cell distribution width; MPV: mean platelet volume; PDW: platelet distribution width; Ig: immunoglobulin; ANA: anti-nuclear antibodies; NAFLD: non-alcoholic fatty liver disease; NASH: non-alcoholic steatohepatitis, ELISA: enzyme-linked immunosorbent assay; HILIC-HPLC: hydrophilic interaction liquid chromatography-high performance liquid chromatography; IF: immunofluorescence; H&E: haemotoxylin and eosin;

Supplementary Table 1. Overview of the investigated traits. Detailed information concerning the different assessment methods is available in the Methods section.

Pathways enriched in diseased samples

Reactome pathways	*Number of Genes*	*P-value*	*FDR*
Activation of C3 and C5 (R-MMU-174577)	4	0.00000588	0.000365
Alternative complement activation (R-MMU-173736)	3	0.00013	0.00699
CD22 mediated BCR regulation (R-MMU-5690714)	14	9.98E-15	1.47E-12
FCGR activation (R-MMU-2029481)	17	1.62E-17	4.37E-15
Scavenging of heme from plasma (R-MMU-2168880)	18	2.06E-18	1.11E-15
Initial triggering of complement (R-MMU-166663)	18	6.64E-18	2.15E-15
Classical antibody-mediated complement activation (R-MMU-173623)	14	4.07E-14	5.48E-12
Role of phospholipids in phagocytosis (R-MMU-2029485)	17	1.27E-16	2.57E-14
Binding and Uptake of Ligands by Scavenger Receptors (R-MMU-2173782)	19	3.07E-18	1.24E-15
Role of LAT2/NTAL/LAB on calcium mobilization (R-MMU-2730905)	13	7.58E-13	7.65E-11
Creation of C4 and C2 activators (R-MMU-166786)	14	1.44E-13	1.67E-11
Regulation of Complement cascade (R-MMU-977606)	21	1.09E-19	1.76E-16
Complement cascade (R-MMU-166658)	21	6.11E-19	4.93E-16
Antigen activates B Cell Receptor (BCR) leading to generation of second messengers (R-MMU-983695)	14	6.55E-13	7.06E-11
FCERI mediated Ca+2 mobilization (R-MMU-2871809)	13	7.20E-12	6.13E-10
FCERI mediated MAPK activation (R-MMU-2871796)	13	1.64E-11	1.33E-09
Regulation of actin dynamics for phagocytic cup formation (R-MMU-2029482)	17	6.47E-14	8.05E-12
Fcgamma receptor (FCGR) dependent phagocytosis (R-MMU-2029480)	19	7.02E-15	1.14E-12
FCERI mediated NF-kB activation (R-MMU-2871837)	13	3.72E-09	2.86E-07
Cell surface interactions at the vascular wall (R-MMU-202733)	19	1.44E-12	1.29E-10
Signaling by the B Cell Receptor (BCR) (R-MMU-983705)	14	6.66E-09	4.68E-07
Fc epsilon receptor (FCERI) signaling (R-MMU-2454202)	13	0.000000133	0.00000894
Peptide ligand-binding receptors (R-MMU-375276)	11	0.0000188	0.00109
Post-translational protein phosphorylation (R-MMU-8957275)	7	0.000869	0.0401
Hemostasis (R-MMU-109582)	33	8.22E-13	7.82E-11

Regulation of Insulin-like Growth Factor (IGF) transport and uptake by Insulin-like Growth Factor Binding Proteins (IGFBPs) (R-MMU-381426)	8	0.000732	0.0348
Innate Immune System (R-MMU-168249)	49	4.62E-16	8.30E-14
Neutrophil degranulation (R-MMU-6798695)	26	6.50E-09	4.78E-07
Class A/1 (Rhodopsin-like receptors) (R-MMU-373076)	14	0.000033	0.00184
Immune System (R-MMU-168256)	67	3.26E-17	7.53E-15
G alpha (i) signalling events (R-MMU-418594)	14	0.000229	0.0116
GPCR ligand binding (R-MMU-500792)	15	0.000198	0.0103
Vesicle-mediated transport (R-MMU-5653656)	23	0.00000511	0.00033
Cytokine Signaling in Immune system (R-MMU-1280215)	14	0.000556	0.0272
Adaptive Immune System (R-MMU-1280218)	24	0.000018	0.00108

Pathways enriched in healthy samples

Reactome pathways	*Number of Genes*	*P-value*	*FDR*
Butyrophilin (BTN) family interactions (R-MMU-8851680)	11	6.44E-06	1.04E-02
CD209 (DC-SIGN) signaling (R-MMU-5621575)	4	8.60E-05	4.63E-02

Supplementary Table 2. Pathways enrichment in healthy vs. disease states

**Components**	**Control diet** (S0587-E001)	**Atherogenic diet** (S0587-E020)
Casein, %	18,600	18,600
Soy protein isolate, %	——	——
Gelatine, %	——	——
Corn starch, pre-gelat., %	40,000	10,000
Maltodextrin, 10 DE, %	13,940	9,690
Sucrose, %	10,000	30,000
pur. Celullose powder, %	5,000	5,000
L-Lysine HCl	——	——
L-Threonine	——	——
L-Tryptophan	——	——
L-Cystine	0,200	0,200
DL-Methionine	0,100	0,100
L-Histidine HCl H2O, %	——	——
Vitamin premix, AIN93G, %	1,000	1,000
Mineral premix, AIN93G, %	3,500	3,500
Choline Cl, %	0,250	0,250
Ca hydrogenphosphate, %	0,400	0,500
BHT, %	0,010	0,010
Cholesterol, %	——	0,150
Butter fat, %	——	20,000
Soybean oil, %	7,000	1,000
Crude protein, %	16,6	16,6
Crude fat, %	7,1	21,0
Crude fibre, %	5,0	5,0
Crude ash, %	3,4	3,5
Starch, %	39,9	9,8
Sugar, %	11,2	31,0
Lysine, %	1,36	1,36
Methionine, %	0,61	0,61
Cystine, %	0,27	0,27
Met + Cys, %	0,88	0,88
Threonine, %	0,72	0,72
Tryptophan, %	0,22	0,22
Arginine, %	0,64	0,64
Histidine, %	0,50	0,50
Valine, %	1,14	1,14
Isoleucine, %	0,93	0,93
Leucine, %	1,63	1,63
Phenylalanine, %	0,85	0,85
Phe+Tyr, %	1,72	1,72

Supplementary Table 3: Composition of diets used in the study

Name of the primer	Sequence (5'-3')
**Microbiome forward primers**	
27F-MID-1	AATGATACGGCGACCACCGAGATCTACACAACCGCATTATGGTAATTGTAGAGTTTGATCCTGGCTCAG
27F-MID-2	AATGATACGGCGACCACCGAGATCTACACAAGGCCTTTATGGTAATTGTAGAGTTTGATCCTGGCTCAG
27F-MID-3	AATGATACGGCGACCACCGAGATCTACACAGAGTGTGTATGGTAATTGTAGAGTTTGATCCTGGCTCAG
27F-MID-4	AATGATACGGCGACCACCGAGATCTACACCACAAGTCTATGGTAATTGTAGAGTTTGATCCTGGCTCAG
27F-MID-5	AATGATACGGCGACCACCGAGATCTACACCGTTCCTATATGGTAATTGTAGAGTTTGATCCTGGCTCAG
27F-MID-6	AATGATACGGCGACCACCGAGATCTACACGCTTGGATTATGGTAATTGTAGAGTTTGATCCTGGCTCAG
27F-MID-7	AATGATACGGCGACCACCGAGATCTACACGTCAACACTATGGTAATTGTAGAGTTTGATCCTGGCTCAG
27F-MID-8	AATGATACGGCGACCACCGAGATCTACACGTCACTGATATGGTAATTGTAGAGTTTGATCCTGGCTCAG
27F-MID-9	AATGATACGGCGACCACCGAGATCTACACTCTCGTCATATGGTAATTGTAGAGTTTGATCCTGGCTCAG
27F-MID-10	AATGATACGGCGACCACCGAGATCTACACTTGGTACGTATGGTAATTGTAGAGTTTGATCCTGGCTCAG
27F-MID-11	AATGATACGGCGACCACCGAGATCTACACCGTTGGATTATGGTAATTGTAGAGTTTGATCCTGGCTCAG
27F-MID-12	AATGATACGGCGACCACCGAGATCTACACCGTTAAGCTATGGTAATTGTAGAGTTTGATCCTGGCTCAG
27F-MID-13	AATGATACGGCGACCACCGAGATCTACACACAGCTCATATGGTAATTGTAGAGTTTGATCCTGGCTCAG
27F-MID-14	AATGATACGGCGACCACCGAGATCTACACGACAAGTGTATGGTAATTGTAGAGTTTGATCCTGGCTCAG
27F-MID-15	AATGATACGGCGACCACCGAGATCTACACGCATTAGCTATGGTAATTGTAGAGTTTGATCCTGGCTCAG
27F-MID-16	AATGATACGGCGACCACCGAGATCTACACTGTGGACTTATGGTAATTGTAGAGTTTGATCCTGGCTCAG
**Microbiome reverse primers**	
338R-MID-A	CAAGCAGAAGACGGCATACGAGATAACCGGAAAGTCAGTCAGCCTGCTGCCTCCCGTAGGAGT
338R-MID-B	CAAGCAGAAGACGGCATACGAGATAGAGTGACAGTCAGTCAGCCTGCTGCCTCCCGTAGGAGT
338R-MID-C	CAAGCAGAAGACGGCATACGAGATCAACTGGTAGTCAGTCAGCCTGCTGCCTCCCGTAGGAGT
338R-MID-D	CAAGCAGAAGACGGCATACGAGATCGTTCGTTAGTCAGTCAGCCTGCTGCCTCCCGTAGGAGT
338R-MID-E	CAAGCAGAAGACGGCATACGAGATCTGTTCACAGTCAGTCAGCCTGCTGCCTCCCGTAGGAGT
338R-MID-F	CAAGCAGAAGACGGCATACGAGATGCTTGCAAAGTCAGTCAGCCTGCTGCCTCCCGTAGGAGT
338R-MID-G	CAAGCAGAAGACGGCATACGAGATGTCAACTGAGTCAGTCAGCCTGCTGCCTCCCGTAGGAGT
338R-MID-H	CAAGCAGAAGACGGCATACGAGATTCCTCATGAGTCAGTCAGCCTGCTGCCTCCCGTAGGAGT
338R-MID-I	CAAGCAGAAGACGGCATACGAGATTCGACTAGAGTCAGTCAGCCTGCTGCCTCCCGTAGGAGT
338R-MID-J	CAAGCAGAAGACGGCATACGAGATTTGCAAGCAGTCAGTCAGCCTGCTGCCTCCCGTAGGAGT
338R-MID-K	CAAGCAGAAGACGGCATACGAGATACACCTCTAGTCAGTCAGCCTGCTGCCTCCCGTAGGAGT
338R-MID-L	CAAGCAGAAGACGGCATACGAGATATCGTAGCAGTCAGTCAGCCTGCTGCCTCCCGTAGGAGT
338R-MID-M	CAAGCAGAAGACGGCATACGAGATCTCTTGACAGTCAGTCAGCCTGCTGCCTCCCGTAGGAGT
338R-MID-N	CAAGCAGAAGACGGCATACGAGATCCTACCATAGTCAGTCAGCCTGCTGCCTCCCGTAGGAGT
338R-MID-O	CAAGCAGAAGACGGCATACGAGATCTGAAGTCAGTCAGTCAGCCTGCTGCCTCCCGTAGGAGT
338R-MID-P	CAAGCAGAAGACGGCATACGAGATACGATCGTAGTCAGTCAGCCTGCTGCCTCCCGTAGGAGT
338R-MID-Q	CAAGCAGAAGACGGCATACGAGATATATGGCCAGTCAGTCAGCCTGCTGCCTCCCGTAGGAGT
338R-MID-R	CAAGCAGAAGACGGCATACGAGATTTCGATGGAGTCAGTCAGCCTGCTGCCTCCCGTAGGAGT
338R-MID-S	CAAGCAGAAGACGGCATACGAGATTACGTACGAGTCAGTCAGCCTGCTGCCTCCCGTAGGAGT
338R-MID-T	CAAGCAGAAGACGGCATACGAGATGATCACGTAGTCAGTCAGCCTGCTGCCTCCCGTAGGAGT
338R-MID-U	CAAGCAGAAGACGGCATACGAGATGTGACAGAAGTCAGTCAGCCTGCTGCCTCCCGTAGGAGT
338R-MID-V	CAAGCAGAAGACGGCATACGAGATTGAGTGTCAGTCAGTCAGCCTGCTGCCTCCCGTAGGAGT
338R-MID-W	CAAGCAGAAGACGGCATACGAGATGAGAAGAGAGTCAGTCAGCCTGCTGCCTCCCGTAGGAGT
338R-MID-X	CAAGCAGAAGACGGCATACGAGATTCTGGACAAGTCAGTCAGCCTGCTGCCTCCCGTAGGAGT

Sequencing primers

16SRead.1.	TATGGTAATTGTAGAGTTTGATCCTGGCTCAG
16SRead.2.	AGTCAGTCAGCCTGCTGCCTCCCGTAGGAGT
16SIndex	ACTCCTACGGGAGGCAGCAGGCTGACTGACT

Mycobiome forward primers

ITSF.SB501	AATGATACGGCGACCACCGAGATCTACACCTACTATATATGGTAATTGGTCCTCCGCTTATTGATATGC
ITSF.SB502	AATGATACGGCGACCACCGAGATCTACACCGTTACTATATGGTAATTGGTCCTCCGCTTATTGATATGC
ITSF.SB503	AATGATACGGCGACCACCGAGATCTACACAGAGTCACTATGGTAATTGGTCCTCCGCTTATTGATATGC
ITSF.SB504	AATGATACGGCGACCACCGAGATCTACACTACGAGACTATGGTAATTGGTCCTCCGCTTATTGATATGC
ITSF.SB505	AATGATACGGCGACCACCGAGATCTACACACGTCTCGTATGGTAATTGGTCCTCCGCTTATTGATATGC
ITSF.SB506	AATGATACGGCGACCACCGAGATCTACACTCGACGAGTATGGTAATTGGTCCTCCGCTTATTGATATGC
ITSF.SB507	AATGATACGGCGACCACCGAGATCTACACGATCGTGTTATGGTAATTGGTCCTCCGCTTATTGATATGC
ITSF.SB508	AATGATACGGCGACCACCGAGATCTACACGTCAGATATATGGTAATTGGTCCTCCGCTTATTGATATGC
ITSF.SB509	AATGATACGGCGACCACCGAGATCTACACCTGAAGTCTATGGTAATTGGTCCTCCGCTTATTGATATGC
ITSF.SB510	AATGATACGGCGACCACCGAGATCTACACACGATCGTTATGGTAATTGGTCCTCCGCTTATTGATATGC
ITSF.SB511	AATGATACGGCGACCACCGAGATCTACACATATGGCCTATGGTAATTGGTCCTCCGCTTATTGATATGC
ITSF.SB512	AATGATACGGCGACCACCGAGATCTACACTTCGATGGTATGGTAATTGGTCCTCCGCTTATTGATATGC
ITSF.SB513	AATGATACGGCGACCACCGAGATCTACACTTGGTACGTATGGTAATTGGTCCTCCGCTTATTGATATGC
ITSF.SB514	AATGATACGGCGACCACCGAGATCTACACCGTTGGATTATGGTAATTGGTCCTCCGCTTATTGATATGC
ITSF.SB515	AATGATACGGCGACCACCGAGATCTACACCGTTAAGCTATGGTAATTGGTCCTCCGCTTATTGATATGC
ITSF.SB516	AATGATACGGCGACCACCGAGATCTACACACAGCTCATATGGTAATTGGTCCTCCGCTTATTGATATGC
ITSF.SB517	AATGATACGGCGACCACCGAGATCTACACGACAAGTGTATGGTAATTGGTCCTCCGCTTATTGATATGC
ITSF.SB518	AATGATACGGCGACCACCGAGATCTACACGCATTAGCTATGGTAATTGGTCCTCCGCTTATTGATATGC
ITSF.SB519	AATGATACGGCGACCACCGAGATCTACACTGTGGACTTATGGTAATTGGTCCTCCGCTTATTGATATGC

Mycobiome reverse primers

ITSR.SA701	CAAGCAGAAGACGGCATACGAGATAACTCTCGAGTCAGTCAGCCGTGARTCATCGAATCTTTG
ITSR.SA702	CAAGCAGAAGACGGCATACGAGATACTATGTCAGTCAGTCAGCCGTGARTCATCGAATCTTTG
ITSR.SA703	CAAGCAGAAGACGGCATACGAGATAGTAGCGTAGTCAGTCAGCCGTGARTCATCGAATCTTTG
ITSR.SA704	CAAGCAGAAGACGGCATACGAGATCAGTGAGTAGTCAGTCAGCCGTGARTCATCGAATCTTTG
ITSR.SA705	CAAGCAGAAGACGGCATACGAGATCGTACTCAAGTCAGTCAGCCGTGARTCATCGAATCTTTG
ITSR.SA706	CAAGCAGAAGACGGCATACGAGATCTACGCAGAGTCAGTCAGCCGTGARTCATCGAATCTTTG
ITSR.SA707	CAAGCAGAAGACGGCATACGAGATGGAGACTAAGTCAGTCAGCCGTGARTCATCGAATCTTTG
ITSR.SA708	CAAGCAGAAGACGGCATACGAGATGTCGCTCGAGTCAGTCAGCCGTGARTCATCGAATCTTTG
ITSR.SA709	CAAGCAGAAGACGGCATACGAGATGTCGTAGTAGTCAGTCAGCCGTGARTCATCGAATCTTTG
ITSR.SA710	CAAGCAGAAGACGGCATACGAGATTAGCAGACAGTCAGTCAGCCGTGARTCATCGAATCTTTG
ITSR.SA711	CAAGCAGAAGACGGCATACGAGATTCATAGACAGTCAGTCAGCCGTGARTCATCGAATCTTTG
ITSR.SA712	CAAGCAGAAGACGGCATACGAGATTCGCTATAAGTCAGTCAGCCGTGARTCATCGAATCTTTG
ITSR.SA713	CAAGCAGAAGACGGCATACGAGATTACGTACGAGTCAGTCAGCCGTGARTCATCGAATCTTTG
ITSR.SA714	CAAGCAGAAGACGGCATACGAGATGATCACGTAGTCAGTCAGCCGTGARTCATCGAATCTTTG
ITSR.SA715	CAAGCAGAAGACGGCATACGAGATGTGACAGAAGTCAGTCAGCCGTGARTCATCGAATCTTTG
ITSR.SA716	CAAGCAGAAGACGGCATACGAGATAACCGGAAAGTCAGTCAGCCGTGARTCATCGAATCTTTG
ITSR.SA717	CAAGCAGAAGACGGCATACGAGATCAACTGGTAGTCAGTCAGCCGTGARTCATCGAATCTTTG
ITSR.SA718	CAAGCAGAAGACGGCATACGAGATCGTTCGTTAGTCAGTCAGCCGTGARTCATCGAATCTTTG
ITSR.SA719	CAAGCAGAAGACGGCATACGAGATCTGTTCACAGTCAGTCAGCCGTGARTCATCGAATCTTTG
ITSR.SA720	CAAGCAGAAGACGGCATACGAGATGCTTGCAAAGTCAGTCAGCCGTGARTCATCGAATCTTTG

ITSR.SA721	CAAGCAGAAGACGGCATACGAGATGTCAACTGAGTCAGTCAGCCGTGARTCATCGAATCTTTG
ITSR.SA722	CAAGCAGAAGACGGCATACGAGATTCCTCATGAGTCAGTCAGCCGTGARTCATCGAATCTTTG
ITSR.SA723	CAAGCAGAAGACGGCATACGAGATTTGCAAGCAGTCAGTCAGCCGTGARTCATCGAATCTTTG
ITSR.SA724	CAAGCAGAAGACGGCATACGAGATACACCTCTAGTCAGTCAGCCGTGARTCATCGAATCTTTG
**Sequencing primers**	
ITSRead.1.	TATGGTAATTGGTCCTCCGCTTATTGATATGC
ITSRead.2.	AGTCAGTCAGCCGTGARTCATCGAATCTTTG
ITSIndex	CAAAGATTCGATGARTCACGGCTGACTGACT

Supplementary Table 4. A list of all primer sequences that were used for NGS sequencing of the 16S rRNA and ITS2 genes in our study.
