# Supplementary material for: Gene-diet interactions associated with complex trait variation in an advanced intercross outbred mouse line
Source: Nat Commun. 2019 Sep 10;10:4097. doi: 10.1038/s41467-019-11952-w (PMC6736984; doi:10.1038/s41467-019-11952-w)
Supplement: Supplementary file 3 — Reporting Summary [file 41467_2019_11952_MOESM3_ESM.pdf]

nature research

Corresponding author(s): Ralf Ludwig

Last updated by author(s): Aug 8, 2019

Reporting Summary

Nature Research wishes to improve the reproducibility of the work that we publish. This form provides structure for consistency and transparency in reporting. For further information on Nature Research policies, see Authors & Referees and the Editorial Policy Checklist.

Statistics

For all statistical analyses, confirm that the following items are present in the figure legend, table legend, main text, or Methods section.

n/a	Confirmed	
☐	☒	The exact sample size (*n*) for each experimental group/condition, given as a discrete number and unit of measurement
☒	☐	A statement on whether measurements were taken from distinct samples or whether the same sample was measured repeatedly
☐	☒	The statistical test(s) used AND whether they are one- or two-sided<br>*Only common tests should be described solely by name; describe more complex techniques in the Methods section.*
☐	☒	A description of all covariates tested
☐	☒	A description of any assumptions or corrections, such as tests of normality and adjustment for multiple comparisons
☐	☒	A full description of the statistical parameters including central tendency (e.g. means) or other basic estimates (e.g. regression coefficient) AND variation (e.g. standard deviation) or associated estimates of uncertainty (e.g. confidence intervals)
☐	☒	For null hypothesis testing, the test statistic (e.g. *F*, *t*, *r*) with confidence intervals, effect sizes, degrees of freedom and *P* value noted<br>*Give P values as exact values whenever suitable.*
☒	☐	For Bayesian analysis, information on the choice of priors and Markov chain Monte Carlo settings
☐	☒	For hierarchical and complex designs, identification of the appropriate level for tests and full reporting of outcomes
☒	☐	Estimates of effect sizes (e.g. Cohen's *d*, Pearson's *r*), indicating how they were calculated

Our web collection on statistics for biologists contains articles on many of the points above.

Software and code

Policy information about availability of computer code

Data collection	The Cast/EJ genome was obtained from: Keane TM, et al. Mouse genomic variation and its effect on phenotypes and gene regulation. Nature 477, 289-294 (2011).
Data analysis	Explained in detail in the Methods section. All code used has been deposited at GitHub (please see Data / Code Availability statements below)

For manuscripts utilizing custom algorithms or software that are central to the research but not yet described in published literature, software must be made available to editors/reviewers. We strongly encourage code deposition in a community repository (e.g. GitHub). See the Nature Research guidelines for submitting code & software for further information.

Data

Policy information about availability of data

All manuscripts must include a data availability statement. This statement should provide the following information, where applicable:

- Accession codes, unique identifiers, or web links for publicly available datasets
- A list of figures that have associated raw data
- A description of any restrictions on data availability

Data Availability
Whole genome sequencing data for BxD2/TyJ, MRL/MpJ and NZM2410/J mice strains has been submitted in database European Nucleotide Archive (ENA) in FASTQ format and publicly available under accession number [PRJEB29771]. The raw sequencing data, i.e. FASTQ files for RNA-Seq, microbiome and mycobiome from NZM2410/J, have been deposited in public database NCBI SRA under accession number [PRJNA543200]. Additionally, Plink formatted genotype data (bed and bim files) for advance inter-cross line mice, quality control of alignment from whole genome sequencing (Qualimap output), VCF files from sequenced strains and founder coefficient plots for every genome-wide QTL is publicly available on the Dryad database [DOI: 10.5061/dryad.c8gc64n]. The data can be visualized and explored at [http://diet.ag-ludwig.com]. The source data underlying Figures 1a, 2b-c, 2e-g, 3a-f, 4a-d, 5a, 5c-f and Supplementary Figures 1a-c and 2a-h, 3a-c, 4a-b are provided as a Source Data file. All other data supporting the findings of this study are contained within the article and its Supplementary information files.

Code Availability
All codes generated or used during the current study are available at Github repository and zenodo database [DOI: 10.5281/zenodo.3347025].

Field-specific reporting

Please select the one below that is the best fit for your research. If you are not sure, read the appropriate sections before making your selection.

☒ Life sciences ☐ Behavioural & social sciences ☐ Ecological, evolutionary & environmental sciences

For a reference copy of the document with all sections, see nature.com/documents/nr-reporting-summary-flat.pdf

Life sciences study design

All studies must disclose on these points even when the disclosure is negative.

Sample size	For the dietary intervention in NZM2410/J mice, a sample size was calculated based on the following assumptions: Primary endpoint: presence of proteinuria, minimum detectable difference in means: 50%, SD: 35%, Groups (3 time points x 3 diets): 9, Power: 80%, alpha: 5%. Using ANOVA an n of 16 / groups is required to detects significant differences.
Data exclusions	No data were excluded
Replication	No replication was performed
Randomization	In all experiemnts randomization was performed by sequential allocation to the different dietary interventions.
Blinding	No blinding was performed because we selected objective endpoints.Sample size

Reporting for specific materials, systems and methods

We require information from authors about some types of materials, experimental systems and methods used in many studies. Here, indicate whether each material, system or method listed is relevant to your study. If you are not sure if a list item applies to your research, read the appropriate section before selecting a response.

Materials & experimental systems

n/a	Involved in the study	
☒	☐	Antibodies
☒	☐	Eukaryotic cell lines
☒	☐	Palaeontology
☐	☒	Animals and other organisms
☒	☐	Human research participants
☒	☐	Clinical data

Methods

n/a	Involved in the study	
☒	☐	ChIP-seq
☒	☐	Flow cytometry
☒	☐	MRI-based neuroimaging

Animals and other organisms

Policy information about studies involving animals; ARRIVE guidelines recommended for reporting animal research

Laboratory animals	Species, strain, sex and age of the laboratory animals is described in the Methods section; in brief: The 4-way advanced intercross line was generated by intercrossing MRL/MpJ, NZM2410/J, BxD2/TyJ and Cast/EiJ strains at equal strain and sex distribution. Mice were intercrossed for 20 generations with at least 50 breeding pairs per generation. For the dietary intervention in experimental lupus, 3-4 week old NZM2410/J at an equal sex distribution were used.
Wild animals	The study did not involve wild animals
Field-collected samples	The study did not involve samples collected from the field
Ethics oversight	Animal experiments for the QTL mapping study were conducted according to the European Community rules for animal care, approved by the respective governmental administration (Ministry for Energy, Agriculture, the Environment and Rural Areas, file number 27-2/13) and performed by certified personnel. Animal experiments with NZM2410/J mice were approved by the Ministry for Energy, Agriculture, the Environment and Rural Areas, file number 35-3/10.

Note that full information on the approval of the study protocol must also be provided in the manuscript.
