# Supplementary material for: Gene-diet interactions associated with complex trait variation in an advanced intercross outbred mouse line
Source: Nat Commun. 2019 Sep 10;10:4097. doi: 10.1038/s41467-019-11952-w (PMC6736984; doi:10.1038/s41467-019-11952-w)
Supplement: Supplementary file 4 — Description of Additional Supplementary Files [file 41467_2019_11952_MOESM4_ESM.pdf]

Description of Additional Supplementary Files

File Name: Supplementary Data 1
Description: Supplementary Data 1 contains a list of genome-wide identified QTL (α_{gw}< 0.05) relevant for figure 1.

File Name: Supplementary Data 2
Description: Supplementary Data 2 contains a list of chromosome-wide identified QTL (α_{cw}< 0.05) relevant for figure 1.

File Name: Supplementary Data 3
Description: Supplementary Data 3 summarizes SNPs and indels data from the whole genome sequencing of the four founder strains used in this study (NZM2410/J, MRL/MpJ, BxD2/TyJ, and CAST/EiJ).

File Name: Supplementary Data 4
Description: Supplementary Data 4 contains a list of differentially expressed genes in spleens of mice when stratified by diet relevant for figure 4.

File Name: Supplementary Data 5
Description: Supplementary Data 5 contains a list of differentially expressed genes in spleens of healthy vs. diseased mice relevant for figure 4.

File Name: Supplementary Data 6
Description: Supplementary Data 6 contains a list of pathways enriched in spleens of mice set on different diets and based on the presence or absence of disease relevant for figure 4.

File Name: Supplementary Data 7
Description: Supplementary Data 7 contains a list of FMC1-associated genes relevant to figure 4.

File Name: Supplementary Data 8
Description: Supplementary Data 8 contains a list of FFC4-associated genes relevant to figure 4.

File Name: Supplementary Data 9
Description: Supplementary Data 9 a list of differentially expressed genes in spleens of ANA positive vs. ANA negative NZM2410/J mice relevant to figure 5.

File Name: Supplementary Data 10
Description: Supplementary Data 10 contains a list of differentially expressed genes in spleens of mice set on calorie-restricted diet vs. NZM2410/J mice set on control and western diets relevant for figure 5.

File Name: Supplementary Data 11
Description: Supplementary Data 11 a list of FMC3-associated genes relevant to figure 5.

File Name: Supplementary Data 12
Description: Supplementary Data 12 contains a list of FMC5-associated genes relevant to figure 5.

File Name: Supplementary Data 13
Description: Supplementary Data 13 contains a list of FFC6-associated genes relevant to figure 5.
